# Post-Meal Responses of Elongation Factor 2 (eEF2) and Adenosine Monophosphate-Activated Protein Kinase (AMPK) to Leucine and Carbohydrate Supplements for Regulating Protein Synthesis Duration and Energy Homeostasis in Rat Skeletal Muscle 

**DOI:** 10.3390/nu4111723

**Published:** 2012-11-13

**Authors:** Gabriel J. Wilson, Christopher J. Moulton, Peter J. Garlick, Tracy G. Anthony, Donald K. Layman

**Affiliations:** 1 Department of Nutritional Sciences, Rutgers University, The State University of New Jersey, New Brunswick, NJ 08901, USA; Email: gabriel.wilson@rutgers.edu (G.J.W.); tanthony@aesop.rutgers.edu (T.G.A.); 2 Division of Nutritional Sciences, University of Illinois at Urbana-Champaign, Urbana, IL 61801, USA; Email: chrismoulton1@gmail.com; 3 Department of Animal Sciences, University of Illinois at Urbana-Champaign, Urbana, IL 61801, USA; Email: pgarlick@ad.uiuc.edu

**Keywords:** translation initiation, translation elongation, branched-chain amino acids, whey protein, mTORC1

## Abstract

Previous research demonstrates that the anabolic response of muscle protein synthesis (MPS) to a meal is regulated at the level of translation initiation with signals derived from leucine (Leu) and insulin to activate mTORC1 signaling. Recent evidence suggests that the duration of the meal response is limited by energy status of the cell and inhibition of translation elongation factor 2 (eEF2). This study evaluates the potential to extend the anabolic meal response with post-meal supplements of Leu or carbohydrates. Adult (~256 g) male Sprague-Dawley rats were food deprived for 12 h, then either euthanized before a standard meal (time 0) or at 90 or 180 min post-meal. At 135 min post-meal, rats received one of five oral supplements: 270 mg leucine (Leu270), 80:40:40 mg leucine, isoleucine, and valine (Leu80), 2.63 g carbohydrates (CHO2.6), 1 g carbohydrates (CHO1.0), or water (Sham control). Following the standard meal, MPS increased at 90 min then declined to pre-meal baseline at 180 min. Rats administered Leu270, Leu80, CHO2.6, or CHO1.0 maintained elevated rates of MPS at 180 min, while Sham controls declined from peak values. Leu80 and CHO1.0 treatments maintained MPS, but with values intermediate between Sham controls and Leu270 and CHO2.6 supplements. Consistent with MPS findings, the supplements maintained elongation activity and cellular energy status by preventing increases in AMP/ATP and phosphorylation of adenosine monophosphate-activated protein kinase (AMPK), acetyl-CoA carboxylase ACC and eEF2. The impact of the supplements on MPS and cellular energy status was in proportion to the energy content within the individual treatments (*i.e.*, Leu270 > Leu80; CHO2.6 > CHO1.0), but the Leu supplements produced a disproportionate anabolic stimulation of MPS, eEF2 and energy status with significantly lower energy content. In summary, the incongruity between MPS and translation initiation at 180 min reflects a block in translation elongation due to reduced cellular energy, and the extent to which Leu or carbohydrate supplements are able to enhance energy status and prolong the period of muscle anabolism are dose and time-dependent.

## 1. Introduction

The rate of skeletal muscle protein synthesis (MPS) is dependent on the availability of energy and amino acids while the anabolic response is modulated by the amino acid leucine (Leu) and the hormone insulin [1] transmitting growth signals via phosphorylation of two nutrient sensors in the cell; namely, the mammalian target of rapamycin complex 1 (mTORC1) and the adenosine monophosphate-activated protein kinase (AMPK) [1,2]. The molecular actions of mTORC1 and AMPK in response to Leu and insulin have been extensively studied in relation to the initiation phase of translation [1,2]; however, the elongation phase of translation has received little attention, particularly in skeletal muscle. 

**Figure 1:**
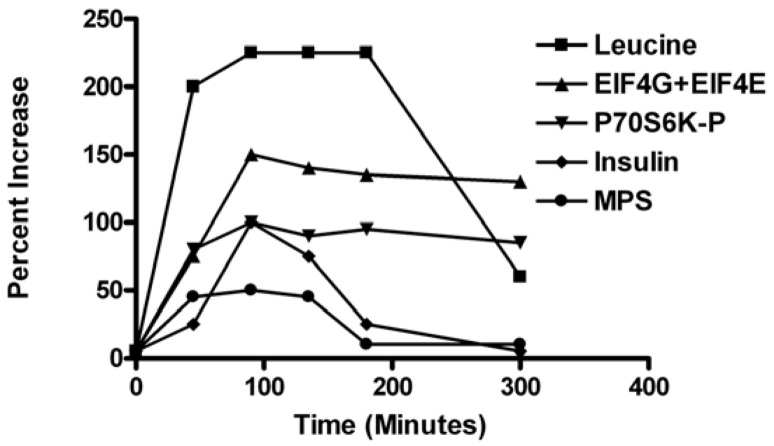
Time course changes in plasma leucine, translation initiation factor p70S6K (active form), Eukaryotic translation initiation factor 4E (active form), eIF4E, plasma insulin, and muscle protein synthesis in gastrocnemius muscle of rats fed a 4-g complete meal containing 20%, 50%, and 30% of energy from whey protein, carbohydrates, and fats, respectively [4].

Previous research characterizing the post-meal time-course of MPS observed rapid stimulation of MPS with peak values around 90 min and a decline to pre-meal baseline by 180 min. The muscle anabolic response was characterized by rapid initiation of MPS associated with increased circulating amino acids and activation of translation factors including phosphorylation of mTORC1 signaling targets eIF4E-binding protein-1 (4E-BP1), the 70-kDa ribosomal protein S6 kinase (S6K1), and assembly of the eukaryotic initiation factor-4F (eIF4F) complex [3,4] (Figure 1). However, the decline in MPS at 180 min post-meal occurred with plasma Leu elevated and initiation factors fully activated (Figure 1). The postprandial decline in MPS was related to a reduced cellular energy status and elongation activity [3]. Oral intake of Leu and/or carbohydrate supplements 2 h after the initial meal served to extend peak rates of MPS by preventing a rise in cellular AMP and phosphorylation of AMPK and elongation factor 2 (eEF2). These findings suggest that the duration of MPS following food intake is in part determined by elongation activity, which in turn is regulated by the energy status of the cell. Considering that protein turnover accounts for more than one quarter of resting energy expenditure [5] and that translation elongation accounts for the majority (>99%) of the energy used for peptide assembly [6,7], it is a logical extension that skeletal muscle would regulate protein synthesis to protect energy status. 

Leu and glucose are both energy substrates in skeletal muscle, but only Leu is a substrate with respect to new protein synthesis and in its catabolism is ketogenic. With these differences in metabolic roles, it seems unlikely that Leu and glucose can similarly extend post-prandial MPS and support cellular energy state. Accordingly, this study sought to define the time and dose dependent effects of Leu and carbohydrate supplements to improve postprandial MPS and cellular energy status when provided as a post-meal supplement. 

## 2. Materials and Methods

*Animals*. Ninety-two male Sprague-Dawley rats (256 ± 8 g; Harlan-Teklad, Madison, WI) were housed individually and maintained at 24 °C with a 12 h reverse light cycle (light period: 19:00–07:00). Rats were fed during the dark period and had free access to water. The animal protocol and facilities were reviewed and approved by the Institutional Animal Care and Use Committee of the University of Illinois at Urbana-Champaign. 

**Figure 2:**
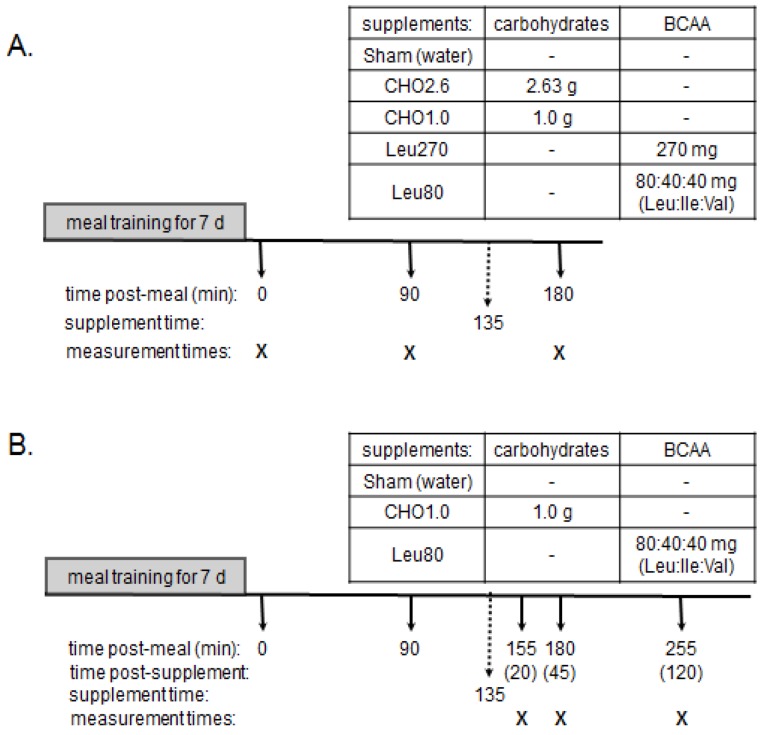
Study Design. Panel A and Panel B represent Experiments 1 and 2, respectively. Both Experiments used a 7-day meal training period with adult male rats (~256 g) receiving 3 meals daily providing 4 g, 4 g, and 6 g of the control diet (Table 1). On day 8, rats received the 4 g breakfast meal at 07:00 (time = 0). Experiment 1 (Panel A) rats were euthanized at times 0 and 90 min as control values for baseline (time 0) and peak (90 min) MPS values. Post-meal supplements were administered by oral gavage at 135 min and treatment groups: Sham, CHO2.6, CHO1.0, Leu270, and Leu80 were euthanized at 180 min. Experiment 2 (Panel B) rats received the Sham, CHO1.0 or Leu80 supplements at 135 min post-meal and were euthanized at 20, 45, or 120 min after the gavage.

*Meal training protocol*. Control diets provided 20%, 50%, and 30%, of energy from protein, carbohydrates, and fats respectively, (Table 1) as previously described [3]. Rats were trained for 7 day to consume 3 meals/day consisting of 4 g meals at 0700 h and 1300 h and a 6 g meal at 1800 h. These meals provided 80% of daily ad libitum intake, which insured that energy intake was equal and that each meal was consumed rapidly (within 20 min of feeding). This minimal food restriction does not alter development of muscle mass but reduces accumulation of body fat [8]. Experimental designs are summarized in Figure 2. 

**Table 1:** Meal composition.

Components	g/kg
Whey Protein Isolate ^1^	1228.0
Corn starch	290.0
Maltodextrin	134.1
Sucrose	101.5
Soybean oil	140.9
Cellulose	53.7
Mineral Mix ^2^	37.6
Vitamin Mix ^2^	10.7
Choline bitautrate	2.7
TBHQ ^3^	0.014

^1^ Whey protein provided by Perham, Perham, MN, USA (89.9% protein, 3.8% carbohydrate, 6.3% other); ^2^ Purchased from Harlen-Teklad, Madison, WI, USA; ^3^ TBHQ, tertiary butylhydroquinone.

***Experiment 1*** evaluated responses of MPS, AMPK, and eEF2 to four energy levels of oral supplements of carbohydrates and BCAA (branched-chain amino acids). On day 8, rats were food deprived for 12 h (19:00–07:00), and then euthanized pre-meal at time 0 (food-deprived, baseline) or at 90 or 180 min after the 4 g control meal. Based on previous research [4], the 90 min time point represented peak MPS activity, while the 180 min time point represented a post-meal time point when MPS declined to pre-meal baseline values. At 135 min post-meal, rats were gavaged with 270 mg Leu (Leu270), 80 mg Leu, 40 mg isoleucine and 40 mg of valine (Leu80), 2.63 g carbohydrates (CHO2.6), 1 g carbohydrates (CHO1.0), or water (Sham control). Animals receiving the supplements were euthanized at 180 min post-meal or 45 min after the gavage.

The CHO2.6 supplement contained 2.63 g of carbohydrates (1.315 g glucose + 1.315 g sucrose; 44.0 kJ) in distilled water, and was designed to be similar to the carbohydrate content of the 4 g control meal (~2.2 g). The CHO1.0 supplement contained 1 g of carbohydrates (0.5 g glucose + 0.5 g sucrose; 16.7 kJ) in distilled water. The Leu270 supplement contained 270 mg of Leu in distilled water (4.5 kJ) equivalent to the daily amount of Leu consumed by rats of this age and strain with free access to AIN-93 diet (Harlan-Teklad, Madison, WI, USA) [9]. The Leu80 supplement contained 80 mg of Leu, 40 mg of isoleucine and 40 mg of valine similar to the balance of BCAA in the control meal (2.7 kJ). The CHO2.6 and Leu270 supplements were based on previous research that produced maximal stimulations of translation initiation and MPS after oral gavage in the fasted state [10,11,12,13]. All gavage treatments were administered in 5 mL.

***Experiment 2*** evaluated temporal changes in MPS, AMPK, and eEF2 at multiple time points after oral supplements to determine the duration of signaling responses. As in Experiment 1, rats were fed the 4 g control meal. At 135 min after the meal, rats received a 5 mL gavage of CHO1.0, Leu80, or water (Sham), as previously described. Rats were sacrificed at 3 time points: 20, 45, and 120 min post-gavage (155, 180, and 255 min post-meal, respectively). These time-points were selected based on previous experiments, and designed to observe maximal anabolic signaling and MPS responses 20 and 45 min after oral gavage, and a decline in MPS and signaling 120 min after the oral bolus [10,11,12,13].

Administration of metabolic tracer and sample collection. MPS was measured in skeletal muscle using the flooding dose method [14]. A 100% enriched L-[^2^H_5_]phenylalanine solution (150 mmol/L; Cambridge Isotopes, Andover, MA) was administered at 150 mmol/100 g body weight and injected via tail vein (1 mL/100 g body weight). After 10 min, animals were euthanized by decapitation, blood was collected in pre-coated EDTA tubes, and hind limbs were quickly removed and immersed in an ice-water mixture. Gastrocnemius muscles were removed from cooled hind limbs, frozen in liquid N_2_, and stored at −80 °C. 

Determination of MPS. Frozen muscle tissue was powdered in liquid nitrogen and protein was precipitated with cold (4 °C) perchloric acid (30 g/L, 1 mL/50 mg muscle tissue). The resulting supernatant and protein pellet were prepared for MPS analysis as described previously [15,16]. The enrichment of L-[^2^H_5_]phenylalanine in the muscle hydrolysate was measured by GC-MS using a 6890N GC and a 5973N mass detector (Agilent Technologies, Santa Clara, CA, USA). Samples were analyzed under electron impact ionization in splitless mode and the mass:charge ratio of phenylethylamine ions at 106 (m + 2) and 109 (m + 5) were monitored for enrichment.

The muscle supernatant was used to determine intracellular free phenylalanine enrichment. Free amino acids were purified by ion exchange resin solid-phase extraction using EZ: faast amino acid analysis sample testing kit (Phenomenex, Torrance, CA, USA) and ^2^H_5_-phenylalanine enrichment was determined using a propyl chloroformate derivative with GC-MS by monitoring the ions at mass:charge ratio 206 (m) and 211 (m + 5) [17]. 

MPS was assessed from the rate of incorporation of L-[^2^H_5_]phenylalanine into total mixed muscle protein as described previously [16]. The time from injection of the tracer until tissue cooling was recorded as the actual time for L-[^2^H_5_] phenylalanine incorporation. MPS, defined as the percentage of tissue protein renewed each day, were calculated according to the formula: MPS = (*E*_b_ × 100)/(*E*_a_ × *t*), where *t* is the time interval between injection and cooling of sampled tissue expressed in days and *E*_b_ and *E*_a_ are the enrichments of [2H5]Phe in hydrolyzed tissue protein and in muscle free amino acids, respectively.

*Plasma measurements*. Plasma was obtained from trunk blood by centrifugation at 1800× *g* for 10 min at 4 °C. Plasma insulin concentrations were analyzed using a commercial RIA kit for rat insulin (Millipore, Billerica, MA, USA). Plasma amino acid concentrations were determined by HPLC using a Waters 2475 Fluorescence detector (Milford, MA, USA) [18]. Plasma glucose was determined by glucose oxidase kit (Invitrogen, Carlsbad, CA, USA).

*Western blot analysis*. Muscle supernatants were subjected to protein immunoblot analysis as previously described [13]. Rabbit polyclonal antibodies were used for total and phospho-AMPKα (Thr172) (adenosine monophosphate-activated protein kinase), total and phospho-ACC (Ser79) (acetyl-CoA carboxylase), total and phospho-eEF2 (Thr56), total and phospho-4E-BP1 (Thr37/46), and total and phospho-Akt (Ser473). All primary antibodies used were purchased from Cell Signaling (Boston, MA, USA), unless stated otherwise. Anti-rabbit IgG, HRP-linked secondary antibody was purchased from Cell Signaling (Boston, MA, USA). 

*Nucleotide analysis*. Adenosine 5′-monophosphate (AMP) and adenosine 5′-triphosphate (ATP) were determined by HPLC analysis as described in technical note CN-039 by Phenomenex (Torrance, CA, USA). Briefly, frozen gastrocnemius muscle was ground under liquid nitrogen and 50 mg of sample was combined with 1 mL of MeOH in water (25:75) and vortexed. This sample was centrifuged at 10,000 RPM at 4 °C for 5 min and the supernatant extracted. The supernatant was dried and then reconstituted in phosphate buffered saline (PBS). The sample was then passed through a Strata X-AW 33u Polymeric Weak Anion Solid Phase Extraction Sorbent (Phenomenex, Torrance, CA, USA). Ten microliter aliquots of the final muscle extract were analyzed by HPLC using a Phenomenex Gemini 5u C18 110A Column 150 × 4.6 mm with temperature set at 25 °C. A single mobile phase was used at a flow rate of 1 mL/min with 25% acetonitrile/10 mM KH_2_PO_4_ pH 7.0 (pH was adjusted with potassium hydroxide (KOH))/5 mM tetrabutylammonium chloride (TBAC). Nucleotide peaks were measured by UV detection at wavelength 260 nm. Peaks were identified by comparison with external standards (Sigma-Aldrich, St. Louis, MO, USA). 

*Statistical analysis*. All data were analyzed by SPSS (version 19) statistical software package for Windows. *Experiment 1* data was analyzed using a one-way ANOVA, with the postprandial times and dietary treatments as the independent variables. When a significant overall effect was detected, differences among individual means were assessed using LSD *post hoc* test. *Experiment 2* data was analyzed using a two-way ANOVA to assess main effects for gavage treatments (Leu80, CHO1.0, and Sham) and time (20, 45, and 120 min post-gavage) as the independent variables. When a significant group × time interaction was detected, differences among treatment groups were assessed with LSD *post-hoc* test. The level of significance was set at *P* < 0.05 for all analysis. Data are reported as mean ± S.E. 

## 3. Results

**Figure 3:**
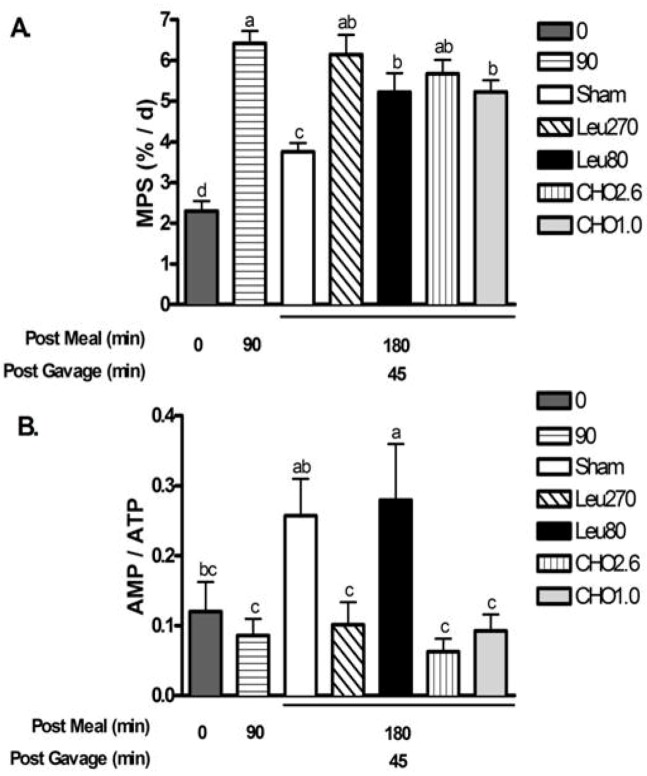
Postprandial changes for muscle protein synthesis (MPS) and AMP/ATP for experiment 1. Values are mean ± S.E.M., *n* = 5–8 per group. Rats were fed a 4 g breakfast meal containing 20% whey protein, and received supplements at 135 min post-meal. Rats were gavaged with water (Sham), 270 mg of leucine (Leu270), 80 mg leucine, 40 mg isoleucine, and 40 mg valine (Leu80), 2.63 g carbohydrates (CHO2.6), or 1 g carbohydrates (CHO1.0). *Panel A* shows changes in muscle protein synthesis (MPS); *Panel B* shows changes in AMP/ATP. Means not sharing a common letter are different (One-way ANOVA *P* < 0.05).

***Experiment 1***. MPS increased at 90 min after meal intake then returned to baseline rates (0 min) by 180 min in the absence of post-meal supplements (Sham) (Figure 3a). Leu270 and CHO2.6 supplements given at 135 min after the meal resulted in MPS rates at 180 min that were similar to 90 min peak values. Supplementation with Leu80 or CHO1.0 also maintained MPS at 180 min above Sham controls but lower than the 90 min peak value (*P* < 0.05). Phosphorylation of AMPKα (Thr172) and ACC (Ser79) (Table 2) and the nucleotide ratio of AMP/ATP (Figure 3b) were measured in muscle as indicators of cellular energy status. Ninety minutes after the control meal, phosphorylation of AMPKα and ACC declined to 56% and 42% of baseline values, respectively, reflecting increased energy availability from the meal. However, ninety minutes later (Sham values at 180 min), phosphorylation of AMPKα and ACC, and the ratio of AMP/ATP significantly increased, consistent with a decline in energy status. All post-meal supplements extended the period of improved energy status but to variable degrees. The CHO2.6, CHO1.0, and Leu270 supplements maintained phospho-AMPKα, phospho-ACC and AMP/ATP ratio at 180 min similar to 90 min. In contrast, Leu80 supplement suppressed phospho-ACC (*P* < 0.05) but not phospho-AMPKα and AMP/ATP ratio was similar to Sham at 180 min. 

**Table 2:** Post-meal signaling ^1^ and plasma insulin ^2^ results for Experiment 1 ^3^.

Time (min) ^4^	AMPK	ACC	eEF2	4E-BP1	Akt	Insulin
0	3.2 ± 0.2 ^a,b^	4.8 ± 0.5 ^a^	4.1 ± 0.1 ^a,b^	1.3 ± 0.2 ^b^	2.3 ± 0.5	43 ± 8.7 ^c^
90	1.8 ± 0.2 ^b^	2.0 ± 0.3 ^b^	2.8 ± 0.3 ^b^	2.5 ± 0.4 ^a^	2.1 ± 0.7	78 ± 5.8 ^a^
180						
Sham ^5^	4.6 ± 0.8 ^a^	4.0 ± 0.7 ^a^	5.1 ± 1.0 ^a^	3.2 ± 0.5 ^a^	1.7 ± 0.2	39 ± 0.4 ^c^
Leu270 ^6^	1.9 ± 0.5 ^c,b^	2.0 ± 0.7 ^b^	2.4 ± 0.2 ^b^	3.3 ± 0.6 ^a^	2.1 ± 0.4	41 ± 1.2 ^c^
Leu80 ^7^	4.8 ± 1.1 ^a^	2.4 ± 0.5 ^b^	3.9 ± 0.8 ^a,b^	3.1 ± 0.5 ^a^	1.7 ± 0.4	40 ± 3.4 ^c^
CHO2.6 ^8^	1.2 ± 0.3 ^c^	1.4 ± 0.4 ^b^	2.3 ± 0.1 ^b^	4.0 ± 0.6 ^a^	2.3 ± 0.4	67 ± 3.2 ^b^
CHO1.0 ^9^	2.6 ± 0.4 ^b^	1.7 ± 0.3 ^b^	2.7 ± 0.5 ^b^	3.9 ± 0.5 ^a^	2.7 ± 0.6	51 ± 3.3 ^b,c^

^1^ Western blot results indicate relative changes in phosphorylation of adenosine monophosphate-activated protein kinase alpha (phospho-AMPKα (Thr172)/total AMPKα), acetyl-CoA carboxylase (phospho-ACC (Ser79)/total ACC), eukaryotic elongation factor 2 (phospho-eEF2 (Thr56)/total eEF2), eIF4E binding protein-1 (phospho-4E-BP1 (Thr37/46)/total 4E-BP1), and phospho-Akt (Ser473)/total Akt; ^2^ Plasma insulin expressed as *pmol/L*; ^3^ Values are means ± S.E.M., *n* = 5–8 per group; means not sharing a common letter are different (One-way ANOVA *P* < 0.05); ^4^ Time expressed as min post-meal; ^5^ Control animals were fed a 4 g breakfast meal containing 20% whey protein, and administered a sham (water) gavage; ^6^ Rats were gavaged with 270 mg of leucine (Leu270); ^7^ Rats were gavaged with 80 mg leucine, 40 mg isoleucine, and 40 mg valine (Leu80); ^8^ Rats were gavaged with 2.63 g carbohydrates (CHO2.6); ^9^ Rats were gavaged with 1 g carbohydrates (CHO1.0).

Phosphorylation of eEF2 (c) was measured as a biomarker of translation elongation (Table 2). Ninety minutes after the meal, eEF2 phosphorylation declined ~30% consistent with increased elongation activity, then significantly increased at 180 min post-meal (Sham) parallel with changes in AMPKα and MPS. The CHO2.6, CHO1.0, and Leu270 supplements reduced eEF2 phosphorylation at 180 min relative to Sham control, while the Leu80 supplement elicited an intermediate response that was not statistically different from Sham control or the 90 min peak value. 

Phosphorylation of 4E-BP1 (Thr37/46) was measured as a biomarker of translation initiation and mTORC1 signaling (Table 2). Consistent with previous findings [3], phosphorylation of 4E-BP1 was higher at 90 min after the control meal and remained elevated at 180 min (Sham) compared with baseline (0 time). This effect was not altered by the provision of post-meal supplements. 

Plasma insulin was elevated at 90 min post-meal and returned to food-deprived (time 0) values at 180 min (Sham) (Table 2). The CHO2.6 and CHO1.0 supplements produced insulin responses intermediate to the fasted (time 0) and fed (90 min) groups, but no differences in phosphorylation of Akt (Ser473) were detected among the groups (Table 2). There were no differences between groups in plasma glucose concentration at 90 or 180 min (data not presented).

Plasma EAAs increased 90 min after the meal and remained elevated at 180 min (Sham) (Table 3). Both leucine supplements further elevated plasma leucine; however, the Leu270 supplement reduced plasma isoleucine and valine whereas the Leu80 supplement increased plasma isoleucine and valine above fasted controls. CHO2.6 and CHO1.0 supplements did not alter plasma amino acids relative to Sham. 

**Table 3:** Post-meal plasma amino acid responses for Experiment 1 ^1,2^.

Time(min) ^3^	Leu	Ile	Val	Lys	Met	Thr
0	163 ± 11 ^d^	123 ± 9 ^b^	192 ± 15 ^c^	743 ± 78 ^b^	80 ± 6 ^b^	726 ± 36 ^b^
90	299 ± 42 ^c^	244 ± 36 ^a^	327 ± 44 ^b^	1016 ± 98 ^a^	122 ± 13 ^a^	955 ± 85 ^a^
180						
Sham ^4^	220 ± 9 ^c,d^	163 ± 7 ^a,b^	256 ± 10 ^b,c^	797 ± 33 ^a,b^	98 ± 7 ^a,b^	780 ± 28 ^a,b^
Leu270 ^5^	1375 ± 113 ^a^	111 ± 11 ^b^	172 ± 16 ^c^	925 ± 68 ^a^	88 ± 5 ^b^	743 ± 47 ^b^
Leu80 ^6^	523 ± 51 ^b^	301 ± 28 ^a^	591 ± 40 ^a^	767 ± 34 ^b^	73 ± 5 ^b^	640 ± 71 ^b^
CHO2.6 ^7^	224 ± 36 ^c,d^	128 ± 18 ^b^	200 ± 37 ^c^	623 ± 117 ^b^	92 ± 11 ^b^	737 ± 51 ^b^
CHO1.0 ^8^	188 ± 22 ^d^	154 ± 20 ^b^	231 ± 27 ^c^	779 ± 80 ^b^	88 ± 13 ^b^	747 ± 42 ^b^

^1^ Plasma amino acids expressed as *μmol/L*; ^2^ Values are means ± S.E.M., *n* = 5–8 per group; means not sharing a common letter are different (One-way ANOVA *P* < 0.05); ^3^ Time expressed as min post-meal; ^4^ Control animals were fed a 4 g breakfast meal containing 20% whey protein, and administered a sham (water) gavage; ^5^ Rats were gavaged with 270 mg of leucine (Leu270); ^6^ Rats were gavaged with 80 mg leucine, 40 mg isoleucine, and 40 mg valine (Leu80); ^7^ Rats were gavaged with 2.63 g carbohydrates (CHO2.6); ^8^ Rats were gavaged with 1 g carbohydrates (CHO1.0).

**Figure 4:**
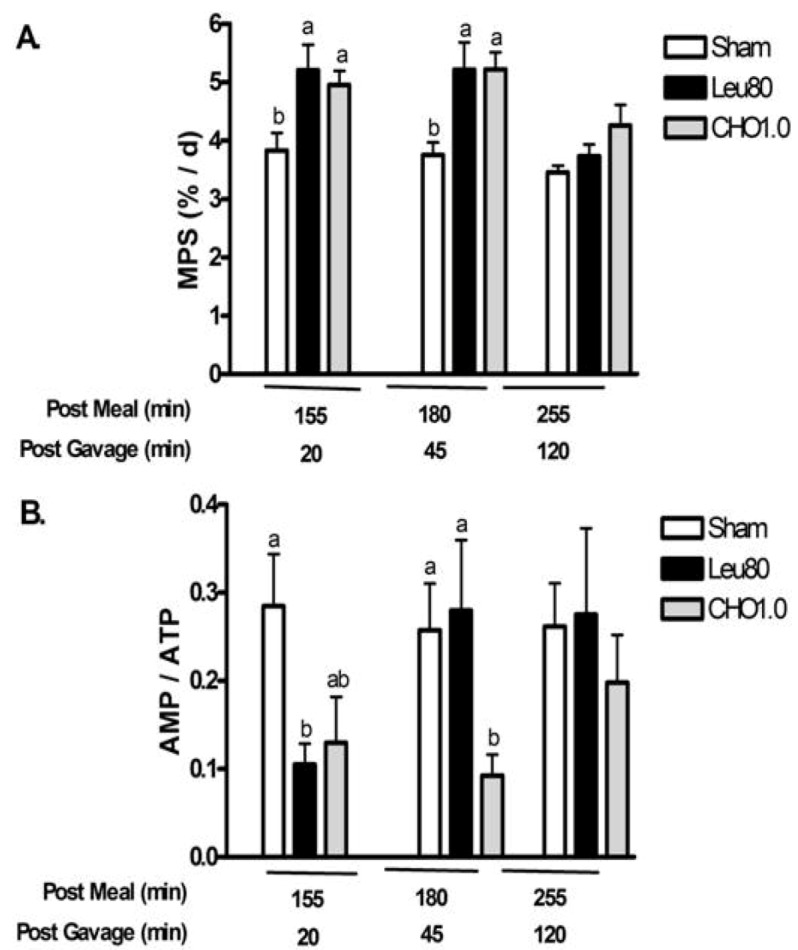
Postprandial changes for muscle protein synthesis (MPS) and AMP/ATP for experiment 2. Values are mean ± S.E.M., *n* = 5–8 per group. Rats were gavaged with water (Sham), 80 mg leucine, 40 mg isoleucine, and 40 mg valine (Leu80), or 1 g carbohydrates (CHO1.0). *Panel A* shows changes in muscle protein synthesis (MPS); *Panel B* shows changes in AMP / ATP. Two-way ANOVA *P* < 0.05 indicated significant main effects for treatment and time, with a significant interaction (*P *< 0.05). Means not sharing a common letter are different within time-points (*P* < 0.05, LSD *post hoc* test).

***Experiment 2***. This study evaluated temporal changes in MPS, AMPK, and eEF2 at multiple time points after oral supplements to determine the duration of signaling responses. At 20 and 45 min post-gavage, both Leu80 and CHO1.0 supplements increased MPS above Sham (two-way ANOVA *P* < 0.05 main effects for treatment and time, with a significant interaction, *P* < 0.05) (Figure 4a). MPS was not different from Sham at 120 min after gavage (255 min post meal). 

Similarly, measurements relating to energy state demonstrated improvements with both supplements at 20 and 45 min post-gavage, with values returning to Sham controls at 120 min. The CHO1.0 treatment tended to reduce phospho-AMPKα (*P* = 0.13) (two-way ANOVA *P* < 0.05 main effect for treatment, time, and GxT interaction) (Table 4) and AMP/ATP (*P* = 0.09) (two-way ANOVA *P* < 0.05 main effect for treatment, time, and interaction) (Figure 4b) relative to Sham control at 20 min post-gavage. The reduction of AMP/ATP, phospho-AMPKα and phospho-ACC (two-way ANOVA *P* < 0.05 main effect for treatment) reached significance at 45 min. All values were similar to Sham controls at 120 min. 

The Leu80 supplement reduced phospho-AMPKα, AMP/ATP, and tended to decrease phospho-ACC (*P* = 0.12) relative to Sham at 20 min post-gavage; however, phospho-AMPKα, and AMP/ATP returned to Sham levels at 45 and 120 min post-gavage for the Leu80 treatment. Phospho-ACC remained depressed 45 min post-gavage for the Leu80 treatment relative to Sham (*P* < 0.05) and returned to Sham values 120 min post-gavage consistent with a temporal sequence for energy signaling. 

The CHO1.0 treatment reduced phosphorylation of eEF2 at 20 and 45 min post-gavage, whereas the Leu80 treatment reduced phospho-eEF2 only at 20 min relative to Sham (two-way ANOVA *P* < 0.05 main effect for treatment and time with a significant interaction) (Table 4). Values were similar across all treatment groups by 120 min. 

**Table 4:** Post-gavage signaling ^1^ and plasma insulin ^2^ results for Experiment 2 ^3^.

Time (min)												
Post Meal		155			180			255				
Post Gavage		20			45			120				
Treatment	Sham ^4^	Leu80 ^5^	CHO1.0 ^6^	Sham ^4^	Leu80 ^5^	CHO1.0 ^6^	Sham ^4^	Leu80 ^5^	CHO1.0 ^6^	Group	Time	G × T
AMPK	4.4 ± 0.3 ^a^	1.8 ± 0.3 ^b^	2.8 ± 0.3 ^a,b,^*	4.6 ± 0.7 ^a^	4.8 ± 1.1 ^a^	2.6 ± 0.4 ^b,^*	4.4 ± 0.8	4.8 ± 0.8	5.0 ± 0.4 *	ys	ys	ys
ACC	3.6 ± 0.6	2.2 ± 0.5 *	2.7 ± 0.4 *	4.0 ± 0.6 ^a^	2.5 ± 0.5 ^b,^*	1.7 ± 0.3 ^b,^*	3.8 ± 0.7	3.4 ± 0.8 *	2.5 ± 0.7 *	ys	ys	ys
eEF2	4.9 ± 0.2 ^a^	2.8 ± 0.7^b^	2.8 ± 0.6 ^b,^*	5.1 ± 1.0 ^a^	3.9 ± 0.7 ^a,b^	2.7 ± 0.5 ^b,^*	4.1 ± 0.6	4.4 ± 0.6	3.6 ± 0.5 *	ys	ys	ys
4E-BP1	2.8 ± 0.2	3.0 ± 0.4	3.8 ± 0.6 *	3.2 ± 0.5	3.0 ± 0.5	3.9 ± 0.5 *	2.9 ± 0.2	3.4 ± 0.5	4.2 ± 0.5 *	ys	ns	ns
Akt	1.8 ± 0.3	2.6 ± 0.4	2.2 ± 0.4	1.7 ± 0.2	1.7 ± 0.4	2.7 ± 0.6	1.8 ± 0.2	2.2 ± 0.3	2.7 ± 0.6	ns	ns	ns
Insulin	42 ± 0.9	41 ± 1.5	47 ± 2.3 *	39 ± 0.4	40 ± 3.4	51 ± 3.3 *	35 ± 0.4	37 ± 4.5	45 ± 0.6 *	ys	ns	ns

^1^ Western blot results indicate relative changes in phosphorylation of adenosine monophosphate-activated protein kinase alpha (phospho-AMPKα (Thr172)/total AMPKα), acetyl-CoA carboxylase (phospho-ACC (Ser79)/total ACC), eukaryotic elongation factor 2 (phospho-eEF2 (Thr56)/total eEF2), eIF4E binding protein-1 (phospho-4E-BP1 (Thr37/46)/total 4E-BP1), and phospho-Akt (Ser473)/total Akt; ^2^ Plasma insulin expressed as pmol/L; ^3^ Values are means ± S.E.M., *n* = 5–8 per group; * Indicates main effect for group (*P* < 0.05). When a group × time (G × T) interaction was significant, differences among treatment groups were assessed with LSD *post-hoc* test. Labeled means without a common letter differ (*P* < 0.05, LSD *post hoc* test); ys = significant *P* < 0.05; ns = not significant *P* < 0.05; ^4^ Control animals were fed a 4 g breakfast meal containing 20% whey protein, and administered a sham (water) gavage at indicated times; ^5^ Rats were gavaged with 80 mg leucine, 40 mg isoleucine, and 40 mg valine (Leu80); ^6^ Rats were gavaged with 1 g carbohydrates (CHO1.0).

**Table 5:** Post-gavage plasma amino acid responses for Experiment 2 ^1, 2^.

Time(min)												
Post Meal		155			180			255				
Post Gavage		20			45			120				
Treatment	Sham ^3^	Leu80 ^4^	CHO1.0 ^5^	Sham ^3^	Leu80 ^4^	CHO1.0 ^5^	Sham ^3^	Leu80 ^4^	CHO1.0 ^5^	Group	Time	G × T
Leu	285 ± 5 ^b^	744 ± 83 ^a,^*	247 ± 13 ^b^	220 ± 8 ^b^	523 ± 51 ^a,^*	188 ± 22 ^b^	173 ± 5	281 ± 22 *	170 ± 7	ys	ys	ys
Ile	192 ± 8 ^b^	482 ± 52 ^a,^*	200 ± 7 ^b^	163 ± 6 ^b^	301 ± 28 ^a,^*	153 ± 20 ^b^	127 ± 4	174 ± 15 *	134 ± 8	ys	ys	ys
Val	301 ± 15 ^b^	703 ± 72 ^a,^*	300 ± 13 ^b^	255 ± 8 ^b^	591 ± 40 ^a,^*	230 ± 26 ^b^	204 ± 7	444 ± 34 *	205 ± 10	ys	ys	ys
Lys	868 ± 68	919 ± 56	894 ± 34	797 ± 29	767 ± 34	778 ± 80	729 ± 25	715 ± 49	783 ± 50	ns	ys	ns
Met	118 ± 10	107 ± 9	108 ± 6	98 ± 6	73 ± 4	88 ± 13	79 ± 5	66 ± 6	84 ± 7	ns	ys	ns
Thr	882 ± 32	721 ± 41 *	722 ± 26	780 ± 23	639 ± 70 *	746 ± 42	673 ± 19	559 ± 87 *	708 ± 76	ys	ys	ns

^1^ Plasma amino acids expressed as μmol/L; ^2^ Values are means ± S.E.M., *n* = 5–8 per group; * Indicates main effect for group (*P *< 0.05). When a group × time (G × T) interaction was significant, differences among treatment groups were assessed with LSD *post-hoc* test. Labeled means without a common letter differ (*P *< 0.05, LSD *post hoc* test); ys = significant *P *< 0.05. ns = not significant *P *< 0.05; ^3^ Control animals were fed a 4 g breakfast meal containing 20% whey protein, and administered a sham (water) gavage at indicated times; ^4^ Rats were gavaged with 80 mg leucine, 40 mg isoleucine, and 40 mg valine (Leu80); ^5^ Rats were gavaged with 1 g carbohydrates (CHO1.0).

4E-BP1 remained in a post-meal hyperphosphorylated state across all treatment groups at all three time points. Further, there was an overall treatment effect for the CHO1.0 supplement to increase phosphorylation of 4E-BP1 (Thr37/46) relative to the Leu80 treatment and Sham control (two-way ANOVA *P* < 0.05 main effect for treatment) (Table 4). 

Plasma insulin was increased by the CHO1.0 supplement relative to Sham and BCAA (two-way ANOVA *P* < 0.05 main effect for treatment) however the magnitude of the change was small (Table 4). While the Leu80 supplement had no effect on plasma insulin relative to Sham. No differences were found for phosphorylation of Akt (Ser473) (Table 4). 

As anticipated, the CHO1.0 supplement had no effect on plasma BCAA relative to sham control (Table 5). On the other hand, the Leu80 supplement increased plasma BCAA above the Sham control or CHO1.0 treatment (two-way ANOVA *P* < 0.05 main effects for treatment and time, with a significant interaction). 

## 4. Discussion

Consistent with our previous findings [3], this study demonstrates that Leu or carbohydrate supplements provided ~2 h after a meal maintains eEF2 activity and extends the postprandial anabolic period of MPS. Further, this study confirms that the reduction in MPS which occurs ~3 h after a meal despite elevated mTORC1 signaling (Figure 2) reflects a block in elongation activity due to reduced energy state and activation of AMPK. The current research emphasizes that the duration of muscle anabolism after a meal is related to cellular energy status, and extends our previous findings by characterizing dose and time-dependent effects to Leu or CHO supplements. 

Supplementation with Leu270 or CHO2.6 at 135 min after the meal maintained peak MPS rate through 180 min (*i.e.*, 45 min post-gavage). Supplementation with Leu80 or CHO1.0 at 135 min post-meal also increased MPS at 180 min above Sham controls; but MPS tended to be lower than the Leu270 and CHO2.6 treatments or the 90 min peak value (Figure 3a). By 120 min post-gavage (255 min post meal), MPS had returned to baseline for both the Leu80 and CHO1.0 treatments and all measures of energy status were similar across the groups. These findings suggest that while the duration of protein synthesis can be extended with supplementation between meals, the magnitude of the effect relates to the specific composition and quantity of the supplement. The implications of these findings to meal feeding behaviors suggest that optimum muscle protein accretion likely relates to precise timing and content of meals and supplements. Indeed, this application is further supported by the findings of Paddon-Jones *et al.* [19] who fed adult humans 3 meals spaced 5 hours apart, and found that adding two supplements of 15 g of essential amino acids (2.79 g of Leu) plus 30 grams of carbohydrates at 2.5 h after the meals increased net protein synthesis for the 16 h period by about 25%. However, long-term feeding studies are needed to investigate the long-term importance of this meal feeding behavior on muscle mass and body composition. 

Similar to our previous study [3], there was an inverse association with measures of energy status (AMP/ATP, phospho-AMPKα, and ACC) and MPS. Proud and colleagues [6,7] reported that inhibition of eEF2 and protein synthesis in response to mild energy deficits using multiple cell lines, is associated with activation of AMPKα, which stimulates the regulatory kinase eEF2K to subsequently phosphorylate (inhibit) eEF2. Further, the ability of leucine to decrease AMPKα phosphorylation has been reported by Du *et al.* [20]. These investigators showed that Leu treatment in C_2_C_12_ myoblast cells resulted in a 36% decrease in the AMP/ATP ratio, a 28% reduction in phospho-AMPKα, and a 43% reduction in AMPK activity. 

The current study demonstrated that both Leu and carbohydrates were effective at maintaining the AMP/ATP ratio and extending post-prandial MPS. The finding that Leu270 (4.5 kJ) and Leu80 (2.7 kJ) supplements were able to maintain energy status (*i.e.*, AMP/ATP, phospho-AMPKα, and ACC) were particularly surprising because of the limited energy contents of the supplements *vs.* CHO2.6 (44.0 kJ) and CHO1.0 (16.7 kJ) treatments. Specifically, the Leu270 with 4-fold less energy content maintains better energy status and greater MPS than CHO1.0 suggesting that oral BCAA supplements are a highly efficient substrate for maintaining post-meal cellular energetics and MPS. These findings may relate to the unique and preferential metabolism of BCAA in skeletal muscle [21,22]. 

The role of Leu in providing energy to skeletal muscle through direct oxidation of the keto acid substrate (α-ketoisocaproate; KIC) is well known, but when compared with the carbohydrate supplements, the magnitude of the energy response to Leu appears to be disproportionately higher than the energy content in the Leu supplement. Supplements of Leu or KIC are also known to produce parallel increases in oxidation of valine and isoleucine. Consistent with this response, we observed depletion of plasma valine and isoleucine with the Leu270 treatment (Table 3). Further, the plasma depletion of valine and isoleucine was blunted with the Leu80 treatment that contained supplemental valine and isoleucine. 

An alternative possibility for the Leu effect on energy status is the potential that Leu has a direct regulatory effect on mitochondrial activity via stimulation of PGC-1α or Sirt-1 [23,24]. Sun *et al.* [23,24] reported that cultured C2C12 myocytes incubated with leucine for 48 h demonstrated increased lipid oxidation, increased oxygen consumption, and increased mitochondrial biogenesis via induction of PGC-1α. Likewise, we have preliminary evidence that prolonged feeding of Leu-rich whey protein meals to adult rats increases gene expression of skeletal muscle PGC-1α, cytochrome B and Tfam as markers of mitochondrial biogenesis [25]. The comparative contribution of Leu to muscle energy status appears to be a novel finding; however, a direct link to mitochondria function remains speculative and requires further investigation. 

The specific energy contents of the 4 treatments also provide unique insight into the temporal changes in energy regulations via AMP/ATP, AMPK, ACC and eEF2. The time course measurements of the Leu80 and CHO1.0 treatments appear to differentiate a hierarchical sequence for energy signaling. Both Leu80 and CHO1.0 treatments maintain MPS at 180 min post-meal (or 45 min post-gavage; Figure 4a). At 20 min after the supplements, the indicators of energy status AMP/ATP (Figure 4b) and phosphorylation of AMPK, ACC, and eEF2 (Table 4) reflect increased energy availability. At 45 min post-gavage, the Leu80 treatment exhibits an increase in AMP/ATP (Figure 4b) and phosphorylation of AMPK (Table 4) similar to the Sham control. The change in energy status is reflected in inhibition of eEF2, but not yet in ACC. Likewise, the inhibition of eEF2 is not yet reflected in MPS (Figure 4a) which represents the net of tracer incorporation for 10 min (from 35 min to 45 min post-gavage. By 120 min post-gavage, MPS returned to baseline for both the Leu80 and CHO1.0 treatments and all measures of energy status were similar across the groups. A similar hierarchical sequence is apparent with Leu80 group in Table 2 with AMPK activated inhibiting eEF2 but ACC has not been affected. Additional research is needed to elucidate energy signaling responses, translation elongation, and post-meal changes in MPS. 

In summary, Leu or carbohydrate supplements provided ~2 h after consumption of a complete meal can extend of the postprandial anabolic period of MPS. This response is associated with maintaining eEF2 activity and cellular energy. However, the novel finding of this research is that the extent to which Leu or carbohydrate supplements is able to enhance energy status and prolong the period of muscle anabolism, are dose and time-dependent. Improved understanding of factors that regulate the duration of MPS may have implications for maintenance of lean tissue during weight loss or aging, for treatment of patients suffering trauma or prolonged bed rest, as well as for acceleration of muscle development in athletes. As such, the roles of eEF2 and AMPK in regulation of postprandial MPS warrant further investigation. 
